# Cobalamin deficiency resulting in a rare haematological disorder: a case report

**DOI:** 10.1186/1752-1947-3-80

**Published:** 2009-10-20

**Authors:** Thomas M Chapuis, Bernard Favrat, Patrick Bodenmann

**Affiliations:** 1 Department of Ambulatory Medicine and Community Healthcare, University of Lausanne, Rue du Bugnon 44, CH-1011 Lausanne, Switzerland

## Abstract

**Introduction:**

We present the case of a patient with a cobalamin deficiency resulting in pancytopaenia, emphasizing the importance to define, diagnose and treat cobalamin deficiency.

**Case presentation:**

A 52-year-old man from the Democratic Republic of Congo presented to the emergency department with shortness of breath and a sore tongue. Physical examination was unremarkable. His haemoglobin was low and the peripheral blood smear revealed pancytopaenia with a thrombotic microangiopathy. The findings were low cobalamin and folate levels, and high homocysteine and methylmalonate levels. Pernicious anaemia with chronic atrophic gastritis was confirmed by gastric biopsy and positive antiparietal cell and anti-intrinsic factor antibodies. Cobalamin with added folate was given. Six months later, the patient was asymptomatic.

**Conclusion:**

Cobalamin deficiency should always be ruled out in a patient with pancytopaenia. Our case report highlights a life-threatening cobalamin deficiency completely reversible after treatment.

## Introduction

Pernicious anaemia is a common cause of cobalamin deficiency, as discussed by Biermer in 1872. Whipple, Minot and Murphy developed a treatment with a liver extract in the late 1920s, suggesting an association between an extrinsic factor found in the liver and an intrinsic factor in the gastric juice. Folkers *et al*. isolated the extrinsic factor, that is, cobalamin, in the late 1940s. In the 1950s, Hodgkin *et al*. used X-ray crystallography to reveal the structure of cobalamin. Nevertheless, there is still no gold standard for diagnosis, and nor are there clear guidelines for the treatment of cobalamin deficiency [1,2].

A population survey in the United States revealed that 1.9% of people older than 60 years of age have undiagnosed pernicious anaemia. The introduction of folate fortification in the United States has left some of the population at risk for a masked cobalamin deficiency [3].

## Case presentation

A 52-year-old man from the Democratic Republic of Congo presented to the emergency department after 14 days of shortness of breath, general weakness, weight loss and a sore tongue. He had no cough, chest pain or fever, and there was nothing significant in his medical and family history. He did not smoke nor drink alcohol. On examination, he appeared uncomfortable, anicteric and afebrile with a respiratory rate of 28 per minute, regular pulse of 70 beats per minute and blood pressure of 111/76 mmHg. A cardiovascular examination showed normal heart sounds and his lungs were clear on auscultation. The patient's tongue was unremarkable, as were the abdominal and neurologic examinations.

**Figure 1:**
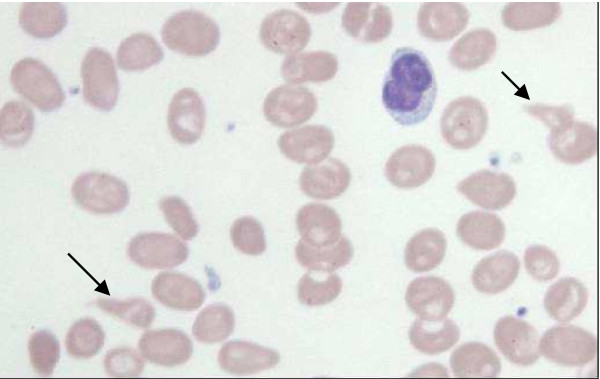
**Peripheral blood smear (May-Grünwald Giemsa stain), 500×**. Macro-ovalocytes. Schistocytes (arrows).

**Figure 2:**
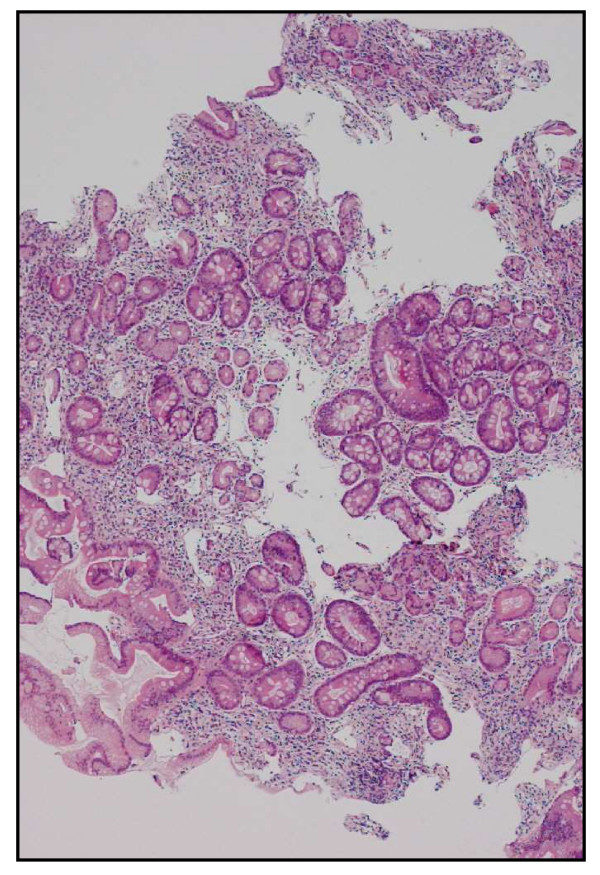
**Gastric (body) biopsy (haematoxylin and eosin), 40×**. Intestinal metaplasia with goblets cells. The lamina propria is filled with lymphocytes.

A chest X-ray and a full blood count were then ordered. The patient's chest X-ray was normal. His full blood count examination showed pancytopaenia. His hematocrit was 21% with a mean corpuscular volume of 107 fl and an absolute reticulocyte count of 31,000/mm^3^. His white blood cell count was 3600/mm^3 ^with 27% neutrophils, 63% lymphocytes and 96,000/mm^3 ^platelets. A peripheral blood smear (Figure 1) revealed anisocytosis and hypersegmented neutrophils. Other findings were low levels of cobalamin (52 pmol/l; normal range: 133-675), folate (4.2 nmol/l; normal value: >6.8), and haptoglobin (<0.1 g/l; normal range: 0.3-2), and high levels of lactate dehydrogenase (LDH) (4604 U/l; normal range: 135-225), homocysteine (Hcys) (101 μmol/l; normal range: 5-15), and methylmalonate (MMA) (1.12 μmol/l; normal value: <0.28). The level of the patient's creatinine (83 μmol/l; normal range: 62-106) and potassium (4.4 mmol/l; normal range: 3.6-4.4) were normal, as were the other findings. A gastroscopy showed oedematous gastric mucosa and a biopsy showed atrophic gastritis (Figure 2). Anti-parietal cell and anti-intrinsic factor antibodies were positive.

The patient was given two units of packed red cells, which improved his respiratory symptoms. Cobalamin (1000 μg intramuscularly) was given daily for 7 days, then weekly for 4 weeks, and then prescribed at a maintenance dose of 1000 μg once a month for life [4]. Daily folate of 5 mg orally was also given for 3 months. The patient was not admitted and follow-up was arranged in our ambulatory setting.

Nine days after introduction of therapy, correction of his deficiencies led to marked reticulocytosis (200,000/mm^3^). Six months later, the patient was asymptomatic and the peripheral blood smear was normal. His levels of cobalamin (505 pmol/l), folate (9.6 nmol/l; >5.3), MMA (0.26 μmol/l; <0.28), and Hcys (9.8 μmol/l; 5-15) were also normal.

## Discussion

Cobalamin deficiency was our leading diagnosis; other causes of pancytopenia including dysthyroidism, HIV, Epstein-Barr virus, cytomegalovirus, herpes simplex virus, hepatitis B virus and hepatitis C virus infection were ruled out afterwards. Myeloproliferative disorders were less likely, and for this reason, a bone marrow aspiration was not performed. Pulmonary tuberculosis was not suggested given the absence of history and a normal chest X-ray. The dramatic response to therapy precluded any necessity for further investigations.

Most patients with cobalamin deficiency exhibit signs of peripheral nervous system or spinal cord involvement [5]. Our patient developed a non-neurologic manifestation - a sore tongue that was proven reversible after treatment. Severe life-threatening haematologic findings such as pancytopaenia, thrombotic microangiopathy and haemolytic anaemia (high LDH, low haptoglobin, schistocytes and thrombopaenia) due to cobalamin deficiency were observed in our patient. These findings are rare and were observed in less than 5% of the patient population in a French study of severe haematological conditions resulting from cobalamin deficiency [5]. There are also case reports involving severe cobalamin deficiency that mimicked leukaemia or thrombotic microangiopathy [6-8].

The prevalence of cobalamin deficiency is notable, especially among older individuals (14%) [9]. General population studies show a prevalence of cobalamin deficiency of around 20% in Western countries [4]. Until recently, it was believed that pernicious anaemia involved mainly Caucasians, but previous studies provide evidence that pernicious anaemia is as common in Africans also [10].

The most common causes of cobalamin deficiency are food-cobalamin malabsorption and pernicious anaemia, mainly among older people. Other causes include dietary deficiency, malabsorption and hereditary metabolic disease [5]. Patients at risk for cobalamin deficiency include those who have had gastric surgery or a history of chronic gastritis, chronic alcoholism, chronic ingestion of antacids, veganism or HIV [4].

Diagnosing cobalamin deficiency is straightforward when the deficiency is profound. Nevertheless, diagnosing a slight deficiency is more difficult in older patients and in patients with neurological symptoms who present a borderline cobalamin level. Classically, the diagnosis is based on a low cobalamin level, but elevated MMA and Hcys levels seem more sensitive and more specific [11]. Renal failure may also increase MMA and Hcys levels and high Hcys level may also reveal a folate deficiency [11].

Solomon evaluated patients for cobalamin deficiency using the standard metabolic markers MMA and Hcys in a retrospective study of the records of patients in an ambulatory setting over a 10-year period [12]. The levels of the three metabolic markers were measured before any treatment was given. Cobalamin, MMA, Hcys levels showed a wide variability over time, and taken individually or in combination did not predict or rule out a haematological or neurological response to cobalamin therapy [12].

**Figure 3:**
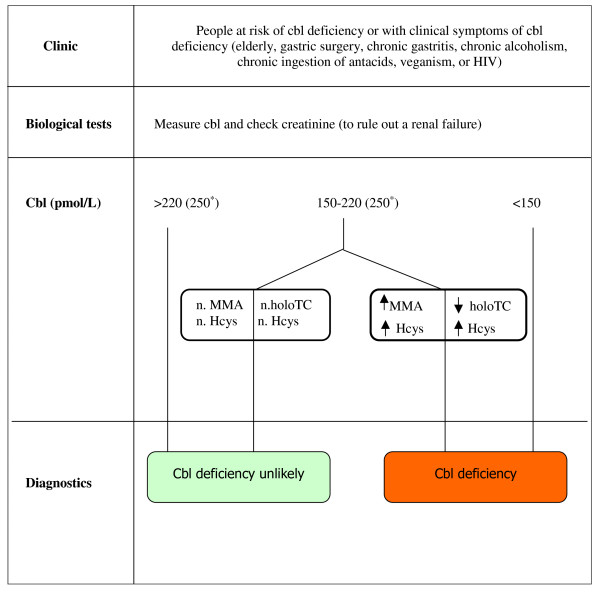
**Diagnostic and therapeutic approach for cobalamin deficiency **[2,4]. Cbl (cobalamin), MMA (methylmalonate), Hcys (homocysteine), holoTC (holotranscobalaminII), PA (pernicious anaemia), n. (normal) *No clear consensus among authors to identify the grey zone (150-250 pmol/L).

There are also new methods available to diagnose cobalamin deficiency. In plasma, total plasma cobalamin is bound to two proteins, haptocorrin and transcobalamin. Normally, 80% of cobalamin is on plasma haptocorrin and forms a holohaptocorrin. About 20% of cobalamin is combined with transcobalamin II to form the biologically active fraction holotranscobalamin II (holoTC), which is available for cell use. HoloTC levels seem to be an early indicator of cobalamin deficiency [13]. Several studies showed that holoTC might be a promising first-line test or used in combination with a metabolic marker (MMA or Hcys) for diagnosing cobalamin deficiency [2,13]. In patients at risk or with clinical symptoms, we suggest a comprehensive care approach (Figure 3) [2,4].

Anti-intrinsic factors are more specific than antiparietal cell antibodies and are found in the serum of almost 40% of patients [14]. Both factors were positive in our patient. A gastric biopsy showed a marked lymphocyte infiltrate in the lamina propria, and the mucosa revealed intestinal metaplasia with goblet cells (Figure 2). The standard treatment of cobalamin deficiency is intramuscular, but oral supplementation has shown an efficacy equal to injection for pernicious anaemia and other cobalamin deficiencies [15]. Life-long cobalamin substitution is necessary in pernicious anaemia [4].

## Conclusion

We present an unusual case of cobalamin deficiency resulting in severe pancytopaenia with thrombotic microangiopathy that was completely reversible after cobalamin substitution. Primary care physicians must be aware of patients at risk for cobalamin deficiencies irrespective of the ethnic origin of the patient. No single algorithm fits every patient, and there is no clear consensus on how to diagnose and treat cobalamin deficiency. Consequently, the diagnosis should rely on clinical epidemiology, clinical symptoms and biochemical markers.

## Abbreviations

Hcys: homocysteine; LDH: lactate dehydrogenase; MMA: methylmalonate.

## Consent

Written informed consent was obtained from the patient for publication of this case report and any accompanying images. A copy of the written consent is available for review by the Editor-in-Chief of this journal.

## Competing interests

The authors declare that they have no competing interests.

## Authors' contributions

TMC carried out the literature review and wrote the case report and is the patient's caregiver. He also obtained the written consent of the patient. BF had a significant role in data analysis and in revising the manuscript. PB had a significant role in data analysis and in the final revision of the manuscript. All authors read and approved the final manuscript.

## References

[B1] Carmel R Green R Rosenblatt DS Watkins D Update on Cobalamin, Folate, and Homocysteine Hematology 2003 62 81 10.1182/asheducation-2003.1.62 14633777

[B2] Schneede J Prerequisites for establishing general recommendations for diagnosis and treatment of vitamin B12 deficiency and cost-utility evaluation of these guidelines Scand J Clin Lab Invest 2003 63 369 376 10.1080/00365510310002068 14599159

[B3] Carmel R Prevalence of undiagnosed pernicious anemia in the elderly Arch Intern Med 1996 156 1097 1100 10.1001/archinte.156.10.1097 8638997

[B4] Andrès E Loukili N Noel E Kaltenbach G Ben Abdelgheni M Perrin A Noblet-Dick M Maloisel F Schlienger J Blicklé J Vitamin B12 (cobalamin) deficiency in elderly patients CMAJ 2004 171 251 259 15289425 10.1503/cmaj.1031155 PMC490077

[B5] Andrès E Affenberger S Zimmer J Vinzios S Grosu D Pistol G Maloisel F Weitten T Kaltenbach G Blicklé J Current hematological findings in cobalamin deficiency. A study of 201 consecutive patients with documented cobalamin deficiency Clin Lab Haematol 2006 28 50 56 10.1111/j.1365-2257.2006.00755.x 16430460

[B6] Dokal I Cox T Galton D Vitamin B12 and folate deficiency presenting as leukaemia BMJ 1990 300 1263 1264 10.1136/bmj.300.6734.1263 2354298 PMC1662842

[B7] Halfdanarson T Walker J Litzow M Curtis H Severe vitamin B12 deficiency resulting in pancytopenia, splenomegaly and leukoerythroblastosis Eur J Haematol 2008 80 5 448 451 10.1111/j.1600-0609.2008.01043.x 18221385

[B8] Garderet L Maury E Lagrange M Najman A Offenstadt Guidet B Schizocytosis in pernicious anemia mimicking thrombotic thrombocytopenic purpura Am J Med 2003 114 423 425 10.1016/S0002-9343(03)00023-8 12714141

[B9] Pennypacker L Allen R Kelly JP Matthews LM Grigsby J Kaye K Lindenbaum K Stabler SP High prevalence of cobalamin deficiency in elderly outpatients J Am Geriatr Soc 1992 40 12 1197 1204 1447433

[B10] Carmel R Ethnic and racial factors in cobalamin metabolism and its disorders Semin Hematol 1999 36 1 88 100 9930571

[B11] Snow C Laboratory diagnosis of vitamin B12 and folate deficiency: a guide for the primary care physician Arch Intern Med 1999 159 1289 1298 10.1001/archinte.159.12.1289 10386505

[B12] Solomon LR Cobalamin-responsive disorders in the ambulatory care setting: unreliability of cobalamin, methylmalonic acid, and homocysteine testing Blood 2005 105 3 978 985 10.1182/blood-2004-04-1641 15466926

[B13] Hvas Am Nexo E Holotranscobalamin-a first choice assay for diagnosing early vitamin B deficiency? J Intern Med 2005 257 289 298 10.1111/j.1365-2796.2004.01437.x 15715686

[B14] Toh B-H Van Driel IR Gleeson PA Pernicious anemia N Engl J Med 1997 337 1441 1448 10.1056/NEJM199711133372007 9358143

[B15] Kuzminski AM Del Giacco EJ Allen RH Stabler SP Lindenbaum J Effective treatment of cobalamin deficiency with oral cobalamin Blood 1998 92 1191 1198 9694707

